# Sensors Technology for Medical Robotics

**DOI:** 10.3390/s22239290

**Published:** 2022-11-29

**Authors:** Víctor F. Muñoz

**Affiliations:** Department of System Engineering and Automation C/Severo Ochoa 4, Universidad de Malaga, 29590 Malaga, Spain; vfmm@uma.es

There are many definitions for the concept of a robot, perhaps too many; it has even been said that we do not know how to define them, but when we see a robot, we identify it. In any case, Tomas Lozano Pérez stated, back in the 1980s, that robotics arose from the intelligent connection between perception and action. The increase in the performance of the intelligent sensing systems allows both movement control loops and human–machine interaction approaches to be much more sophisticated. Thus, the development of intelligent sensors became very important for robotics applications outside of classical industrial environments. Indeed, the use of robots in unstructured environments requires the design and development of sensory systems able to obtain as much information as possible from the environment. It is necessary to provide robotics systems with the required data so that they can carry out their decision-making processes. A paradigm of these unstructured scenarios, which also involves intense human–robot collaboration, is medical applications. Sensor technology can be applied to medicine in the steps of diagnosis and treatment. In this way, intelligent sensory systems are often used for assisting in diagnosis activities and, combined with robots, are usually used for surgery and rehabilitation treatments. Thus, this Special Issue begins with papers devoted exclusively to the use of sensors for diagnosis, and then moves on to papers on the use of robots in medical applications.

Thus, with regard to the diagnosis, the work developed by Nagae et al. [1] presents a smart sensor, documented with trials, capable of detecting sadness, anger, surprise, and joy by using resistive changes in the skin. This system is useful for identifying the emotions of people with autistic syndrome disorder under treatment in order to design an accurate therapy. This smart sensor provides very useful information to the specialist for use in treatment. The second paper, dedicated to the use of sensors to assist in diagnosis, is presented by Ceccarelli et al. [2]. The paper presents a wearable device for monitoring the breathing rate of the wearer. It is a system that combines accelerometers to detect the movement of the ribs during breathing activity to help detect postoperative complications.

The COVID-19 pandemic revitalized the applications of robotics by deploying them in tasks such as disinfection, in-hospital logistics, and tele-operated device management in infectious areas. The latter application is addressed in the work by Li et al. [3], where a master–slave robotic system has been adapted for the use of an ultrasound probe. The contribution of this work focuses on a shared control scheme that is guaranteed to move the probe, in an appropriate way, over a tissue. It uses audio feedback instead of haptic feedback and presents a study on two different types of scenarios. Regarding the use of robots in minimally invasive surgery (MIS), some of the commercial systems lack haptic feedback. The work of Park et al. [4] proposes a device consisting of a force sensor that is attached to the body of the surgical tool carried by the robot. A second device, which receives the information from the force sensor, is placed on the teleoperative mechanism to provide haptic sensation to the operating specialist. The paper presents a calibration of the haptic feedback of this device for different types of tissues. Another approach for haptic feedback proposed by the same author results from the use of a viscoelastic device that changes its properties thanks to a magnetic field [5]. In this case, the surgeon receives haptic feedback through a hemisphere of this material that emulates the properties of the tissue interacting with the surgical instrument equipped with the force sensor.

Another challenge of MIS is the so-called intravascular interventional surgery (VAS). These kinds of procedures use catheters that navigate through blood vessels and are designed, among other tasks, for heart valve replacement. In this procedure, the surgeon guides the catheter from an entry port in a blood vessel to its destination. Although guidance is provided using X-rays during the procedure, it is of interest to understand the forces exerted by the catheter head on the blood vessel wall to avoid damaging it. In this way, the paper by Bandari et al. [6] proposes a force sensor based on the use of an optical fibre array. This is placed on the catheter head, and the deformation of the optical fibre array caused by the contact forces is used for estimating the interaction forces. Another form of haptic assistance is the provision of information about the tension forces of surgical thread during a laparoscopic suturing procedure. For this purpose, Jung et al. [7] propose the use of a deep learning neural network that analyses the images provided by an endoscopic camera.

Intelligent sensory systems in surgery provide the surgeon with additional decision-useful data, as discussed above, through haptic feedback. This information can be included within the motion control loop of a robotic system to assist the surgeon in surgical maneuvers which involve both precision and complexity. For this purpose, Muñoz et al. [8] propose the use of a master–slave system that uses shared control and haptic feedback to assist the surgeon in endonasal surgery procedures. It specializes in replicating safe surgical instrument movements performed by the surgeon in a teleoperated scheme with virtual fixtures, while the additional robotic arm is able to collaborate with other surgical tools in an autonomous way.

The field of rehabilitation is another area where sensory systems and robotics play an important role. This is shown in the paper written by De la Torre et al. [9], where a literature review of works related to the use of robotics in upper limb rehabilitation is presented. On the other hand, Cardoso et al. [10] show the use of brain–computer interfaces (BCI) as a support to lower limb rehabilitation with a passive pedaling device. The authors show an experiment in which the information provided by the BCI is used when volunteers perform the imaginative movement of pedaling. These data are used to move a motor that drives the pedals. To conclude this Special Issue, Diez et al. [11] propose a novel use of a handheld exoskeleton beyond its use for rehabilitation. Specifically, they propose its use to provide a stable hand grip to volunteers who have to remain upright despite disturbances that alter their state of balance.

In conclusion, this Special Issue presents a collection of innovative works in the application of sensors and robotics in the field of medicine. Works on diagnostics, haptic feedback in surgery, surgical robotics, and applications of devices for upper and lower limb rehabilitation have been presented. I hope that some of this work will inspire readers in their future research.
